# *Wolbachia* infection in field-collected *Aedes aegypti* in Yunnan Province, southwestern China

**DOI:** 10.3389/fcimb.2022.1082809

**Published:** 2022-11-30

**Authors:** HengDuan Zhang, Jian Gao, Zu Ma, Yuan Liu, Ge Wang, Qing Liu, YuTong Du, Dan Xing, ChunXiao Li, Teng Zhao, YuTing Jiang, YanDe Dong, XiaoXia Guo, TongYan Zhao

**Affiliations:** State Key Laboratory of Pathogen and Biosecurity, Beijing Institute of Microbiology and Epidemiology, Beijing, China

**Keywords:** *Aedes aegypti*, *Wolbachia*, *wsp* gene, phylogenetics, China

## Abstract

**Background:**

*Wolbachia* is gram-negative and common intracellular bacteria, which is maternally inherited endosymbionts and could expand their propagation in host populations by means of various manipulations. Recent reports reveal the natural infection of *Wolbachia* in *Aedes Aegypti* in Malaysia, India, Philippines, Thailand and the United States. At present, none of *Wolbachia* natural infection in *Ae. aegypti* has been reported in China.

**Methods:**

A total of 480 *Ae. aegypti* adult mosquitoes were collected from October and November 2018 based on the results of previous investigations and the distribution of *Ae. aegypti* in Yunnan. Each individual sample was processed and screened for the presence of *Wolbachia* by PCR with *wsp* primers. Phylogenetic trees for the *wsp* gene was constructed using the neighbour-joining method with 1,000 bootstrap replicates, and the p-distance distribution model of molecular evolution was applied.

**Results:**

24 individual adult mosquito samples and 10 sample sites were positive for *Wolbachia* infection. The *Wolbachia* infection rate (IR) of each population ranged from 0 - 41.7%. The infection rate of group A alone was 0%-10%, the infection rate of group B alone was 0%-7.7%, and the infection rate of co-infection with A and B was 0-33.3%.

**Conclusions:**

*Wolbachia* infection in wild *Ae. aegypti* in China is the first report based on PCR amplification of the *Wolbachia wsp* gene. The *Wolbachia* infection is 5%, and the *wAlbA* and *wAlbB* strains were found to be prevalent in the natural population of *Ae. aegypti* in Yunnan Province.

## Introduction

*Wolbachia* are common gram-negative intracellular bacteria that are maternally inherited endosymbionts and can propagate in host populations *via* various manipulations. *Wolbachia* was first discovered in the reproductive tissue of *Culex pipiens pipiens* in 1924 (Hertig, 1924) and later found in field-collected mosquitos *(* Carvajal et al., 2019 *)*. It is estimated to naturally occur in 66% of known insect species, including fruit flies, mosquitos, tsetse flies, bed bugs, ants, kissing bugs, and termites (Hilgenboecker et al., 2008; Werren et al., 2008; Beckmann et al., 2017).

The ecological interactions between *Wolbachia* and its eukaryotic host cells cover a wide range, including parasitism, symbiosis, and reciprocity (Werren et al., 2008; Hosokawa et al., 2010; Inácio da Silva et al., 2021). Because of the unique ability of *Wolbachia* to infect and manipulate the reproductive mode of the host, it has deeply influenced not only the ecology and evolution of its host but also the host’s reproductive biology through extensive symbiosis (Landmann, 2019; Ding et al., 2020). The effects of *Wolbachia* on the reproductive mode of the host mainly include the induction of cytoplasmic incompatibility (CI), parthenogenesis, male feminization, and male-killing increases in male mortality (Werren, 1997). In addition, *Wolbachia* induces CI during the fusion of male and female gametes (Ross et al., 2019), which not only suppresses mosquito populations but also inhibits the replication of viruses and parasites within mosquitoes, such as dengue virus (DENV), chikungunya virus (CHIKV), yellow fever virus (YFV), Zika virus (ZIKV) and *Plasmodium* parasites (Moreira et al., 2009; Bian et al., 2010; Walker et al., 2011; van den Hurk et al., 2012; Aliota et al., 2016; Ahmad et al., 2017).

Before 2014, natural *Wolbachia* infections were mainly concentrated in the *Cx. pipiens* complex and in *Ae. Albopictus* (Song She-Wu, 2002a; Song She-Wu, 2002b), and no natural infection was found in *Ae. aegypti* and *Anopheles* (Cui Bei-jin, 2015). However, natural *Wolbachia* infections were recently found in *Anopheles gambiae* in Burkina Faso, Mali, and areas of West Africa. In addition, *Wolbachia* infections in *Ae. aegypti* were found in Malaysia, India, the Philippines, Thailand, and the United States (Baldini et al., 2014; Teo et al., 2017; Hegde et al., 2018; Thongsripong et al., 2018; Balaji et al., 2019; Bennett et al., 2019; Carvajal et al., 2019; Kulkarni et al., 2019). At present, no natural *Wolbachia* infections in *Ae. aegypti* have been reported in China. *Ae. aegypti* is distributed in southern provinces in China, such as Hainan and Guangdong Provinces. Since the first discovery of *Ae. aegypti* in 2002 at Ruili Port in Yunnan Province, *Ae. aegypti* larvae and adults have been collected in 9 cities across Yunnan Province, indicating a rapid invasion and spread of this species (Shi et al., 2017). This rapid spread is highly concerning given that *Ae. aegypti* plays an important role in the transmission of the dengue virus and other mosquito-borne diseases and that *Wolbachia* infections in *Ae. aegypti* are linked to multiple invasions. Thus, this study investigated natural *Wolbachia* infection in this species in the field, especially in the border areas along Yunnan Province, which are the location of the invasion and spread of *Ae. aegypti*.

The common genes used to detect *Wolbachia* infection in a host species with polymerase chain reaction (PCR) include *wsp* (*Wolbachia* surface protein), *ftsZ* (filamenting temperature-sensitive mutant Z) and *16S* rRNA. Genetic drift in *ftsZ* and *16S* rRNA genes is low; thus, they can be used for stable amplification and classification of partial sequences with large differences at the species level. However, the highly variable marker gene *wsp* has a very similar genetic relationship, yet evolves faster than the former two and thus cannot be used to distinguish between species. Instead, it is easier to type closely related *Wolbachia* to determine the phylogenetic relationship of *Wolbachia* in greater detail (Braig et al., 1998; Zhou et al., 1998a). With the use of PCR and sequencing techniques, *Wolbachia* has been divided into 17 groups (A-Q) (Augustinos et al., 2011; Wang et al., 2014; Glowska et al., 2015; Wang et al., 2016). Groups A and B are typically capable of reproductive manipulation and are mainly distributed in arthropods (Werren et al., 1995; Ellegaard et al., 2013).

*Wolbachia* as a new technology to control mosquito and mosquito-borne diseases is more long-lasting and environmentally friendly than traditional insecticide methods. By releasing *Wolbachia*-infected mosquitoes into target areas, the control of mosquito and mosquito-borne diseases has been applied in the United States, Australia and Mexico (Hoffmann et al., 2011; O’Neill, 2018; Mains et al., 2019; Che-Mendoza et al., 2021; Utarini et al., 2021). *Ae. aegypti* is the main transmission vector of dengue fever in Yunnan Province which is one of the main provinces for dengue fever outbreaks. With the increase of mosquito resistance, it is important to develop new protection methods. It is important to realize the infection status and types of *Wolbachia* in major vectors in the region, in order to evaluate the application of Wolbachia in the future. Therefore, this study aimed to evaluate natural *Wolbachia* infections in *Ae. aegypti* collected from different sites in Yunnan Province using *Wolbachia wsp* gene amplification to detect and type the infection.

## Materials and methods

### Description of the study area

Yunnan Province is located in southwestern China that comprises 16 prefectures and 129 counties, and extends from 21°8′32″ to 29°15′8″N and 97°31′39″ to 106°11′47″E which shares a 4,060-km border with Laos, Vietnam, and Myanmar. The climate in most regions of this province is fairly mild in winter and rather cool in summer. The temperature information of the sampling site was collected as shown in **Table 1**.

**Table 1:** Temperature of sampling areas in Yunnan Province, 2018.

Collection regions	City/Month	AHT(°C)	EHT(°C)	ALT(°C)	ELT(°C)
Xishuangbanna prefecture	JH/10 month	29	33	20	16
	MH/10 month	24	28	15	13
	ML/10 month	28	32	19	16
Dehong Prefecture	RL/10 month	27	30	18	12
	RL/11 month	26	28	12	8

AHT, The average high temperature; EHT, The Extreme high temperature; ALT, The average low temperature; ELT, The Extreme low temperature; JH, Jionghong City; MH, Menghai Country; ML, Menglai Country; RL, Ruili City.

### Mosquito sampling and DNA isolation

Samples were collected in July-August 2017, October-November 2018, and September 2019 and 2020. Samples were collected in the months in which *Ae. aegypti* breed in Yunnan, and the sample collection periods were consecutive. As the samples of *Ae. aegypti* in 2017, 2019 and 2020 were negative and positive only in the samples of 2018.

In the present study, 19 populations were collected between October and November 2018 based on the results of previous investigations and the distribution of *Ae. aegypti* in Yunnan Province (**Table 2**). Larvae were collected and reared to adults. All larvae collected from one breeding container were stored into a bottle, a bottle represents a breeding container. These bottles were brought back to the laboratory for eclosion. According to the standard of the people’s Republic of China: NY/T1964.3-2010, adult mosquitoes and larvae were cultured in 26±1°C and 75±10% humidity in lab. Adult mosquitoes were fed with 8% sugar water, and larval were fed with powder which implement the national standard GB 14924.3-2010 (crude protein≥ 20%, crude fat content≥ 4%, crude fiber content ≤5%). All strains *of Aedes aegypti* in our insectary have fed with this diet all the time and *Wolbachia* free. Adult female mosquitoes after 7 days of eclosion were morphologically identified and COI identification and then for *Wolbachia* detection. According to the sample collection records, the different containers included waste tires, buckets, flowerpot, hydroponic plants and water basins. The distribution of *Ae. aegypti* in Yunnan is mainly concentrated in Xishuangbanna, Dehong and Lincang, although there are reports of distribution in other counties in Yunnan, but no local *Ae. aegypti* has been reported for several years. DNA isolation was conducted with the QIAamp^®^ Fast DNA Tissue Kit (Qiagen, Dusseldorf, Germany) according to the manufacturer’s protocol, and all the DNA samples were stored at -80°C (Jiao Jun, 2022).

**Table 2:** Collection information of *Ae. aegypti* in Yunnan Province in 2018.

Collection region	No.	Collection site	Size	Coordinates	Collection time
Xishuangbanna prefecture	YA1	JHMJ	30	N22°00’04’’, E 100°48’26’’	10/28/2018
	YA2	JHGZ	12	N22°00’38’’, E 100°49’07’’	10/26/2018
	YA3	JHDM	30	N21°58’09’’, E 100°47’47’’	10/25/2018
	YA4	JHMG	30	N22°00’38’’, E100°49’07’’	10/26/2018
	YA5	JHFZ	13	N21°59’60’’, E100°47’13’’	10/28/2018
	YA6	DLYL	15	N21°40’52’’, E100°02’06’’	10/27/2018
	YA7	JHML	30	N21°29’16’’, E101°34’04’’	10/25/2018
	YA8	JHGL2	30	N22°00’04’’, E100°48’26’’	10/28/2018
	YA9	DLAA	30	N21°40’49’’, E100°02’10’’	10/27/2018
	YA10	DLXX	30	N21°44’46’’, E100°11’07’’	10/27/2018
	YA11	JHML	30	N21°59’24’’, E100°48’41’’	10/27/2018
	YA12	JHJL	12	N22°00’20’’, E100°47’55’’	10/26/2018
Dehong Prefecture	YA13	JGTA	30	N23°58’53’’, E097°53’14’’	11/01/2018
	YA14	JGLS	30	N23°59’54’’, E097°53’14’’	11/01/2018
	YA15	JGYH	8	N23°57’53’’, E097°53’14’’	11/01/2018
	YA16	RLHP	30	N24°00’42’’, E097°51’08’’	11/02/2018
	YA17	RLJK	30	N23°59’08’’, E097°52’31’’	10/31/2018
	YA18	RLPP	30	N24°00’18’’, E097°52’58’’	11/01/2018
	YA19	RLHF	30	N24°00’26’’, E097°52’53’’	11/01/2018

### Molecular identification of mosquitoes

The DNA samples that were morphologically identified as *Ae. aegypti* were subjected to molecular identification of the COI gene to ensure that the experimental mosquitoes were *Ae. aegypti* (Bonacum et al., 2001).

### Detection of *Wolbachia* infection

Following the *Wolbachia wsp* gene classification method established by Zhou et al. (Zhou et al., 1998b), three pairs of diagnostic primers were selected to detect and identify *Wolbachia*. The downstream primer was 691R (AAAAATTAAACGCTACTCCA), and the upstream primers were 81F (TGGTCCAATAAGTGATGAAGAAAC); 328F (CCAGCAGATACTATTGCG) and 183F (AAGGAACCGAAGTTCATG). 81F is a universal primer that can amplify a fragment of approximately 590-632 bp in all known *Wolbachia* strains. 328F amplifies a fragment of approximately 380 bp that is specific to *Wolbachia* Group A, whereas 183F amplifies a fragment of approximately 501 bp that is specific to *Wolbachia* Group B. The volume of amplified PCR was 50 µL, including 25 µL of Taq DNA polymerase, 16 µL of double-distilled H_2_O, 2 µL of upstream and downstream primers at a concentration of 10 μmol/L, and 5 µL of the DNA template. The amplification conditions were 95°C predenaturation for 3 min, then [amplification at 94°C for 1 min, 55°C for 1 min, 72°C for 1 min] repeated for 35 cycles with a final extension of 72°C for 7 min. Five microlitres of the above PCR product was used in a 1.2% agarose gel electrophoresis, the results of which were examined under UV light, and the remainder was sent to Tianyi Biotechnology Company, Ltd. for sequencing.

### Phylogenetic analysis

All aligned *Wolbachia* sequences were compared with other sequences available in the GenBank database to determine the percentage identity using BLAST (http://blast.ncbi.nlm.nih.gov/Blast.cgi). The most similar sequences were downloaded for phylogenetic analysis. The selected sequences of *Wolbachia* strains (**Table S1**; **Table S2**; **Table S3**) and those obtained in the study then underwent multiple alignments using Clustal W 2.0.10. A phylogenetic tree for the *wsp* gene was constructed using the neighbour-joining method with 1,000 bootstrap replicates, and the p-distance distribution model of molecular evolution was applied.

## Results

### *Ae. aegypti* identification

Morphological identification and molecular identification of the COI gene determined that all 480 mosquitoes in this study were *Ae. aegypti.* All sequences have been submitted to the GenBank database with accession numbers ON637917 to ON637937.

### Detection of *Wolbachia* in mosquitoes

**Figure 1:**
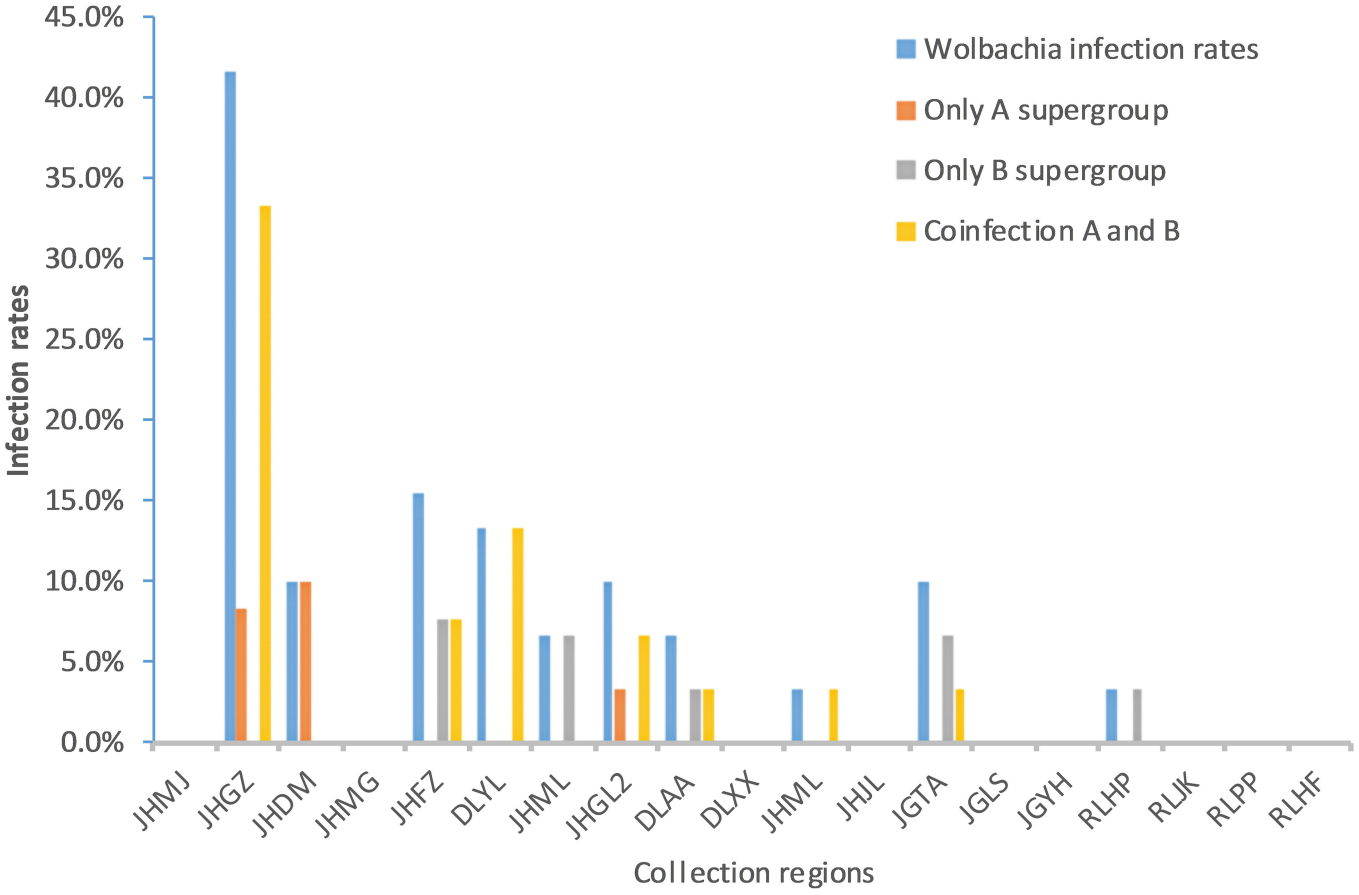
Comparison of *Wolbachia* infection rates among different collection regions in Yunnan Province. JHMJ、JHGZ、JHDM、JHMG、JHFZ、DLYL、JHML、JHGL2、DLAA、DLXX、JHML and JHJL belong to Xishuangbanna prefecture, Yunnan. JGTA、JGLS、JGYH、RLHP、RLJK、RLPP and RLHF belong to Dehong prefecture, Yunnan.

Fragments of 500 bp and 380 bp were amplified with *Wolbachia* A- and *Wolbachia* B-specific primers, respectively, from each of the 30 samples from each *Ae. aegypti* population, confirming *Wolbachia* infection. The sequencing results showed that 24 (5%) adult mosquito samples were positive for *Wolbachia* infection (**Table 3**). These individuals were collected from 10 sites: 8 in Xishuangbanna Prefecture and 2 in Dehong Prefecture. The *Wolbachia* infection rate (IR) of each population ranged from 0% to 41.7%. The infection rate of Group A alone was 0–10%, the infection rate of Group B alone was 0–7.7%, and the rate of coinfection with Groups A and B was 0–33.3% (**Figure 1**; **Table 3**).

**Table 3:** Infection of *Ae. aegypti* with *Wolbachia* in Yunnan Province.

Collection regions	Code	Numbers	*Wolbachia*			*Wolbachia* A		*Wolbachia* B		*Wolbachia* A & B	
			numbers	rate	95% CI	numbers	rate	numbers	rate	numbers	rate
Xishuangbanna prefecture	JHMJ	30									
	JHGZ	12	5	41.70%	0.138 ~ 0.696	1	8.30%			4	33.30%
	JHDM	30	3	10.00%	-0.007 ~ 0.207	3	10.00%				
	JHMG	30									
	JHFZ	13	2	15.40%	-0.042 ~ 0.350			1	7.70%	1	7.70%
	DLYL	15	2	13.30%	-0.039 ~ 0.305					2	13.30%
	JHML	30	2	6.70%	-0.023 ~ 0.156			2	6.70%		
	JHGL2	30	3	10.00%	-0.007 ~ 0.207	1	3.30%			2	6.70%
	DLAA	30	2	6.70%	-0.023 ~ 0.156			1	3.30%	1	3.30%
	DLXX	30									
	JHML	30	1	3.30%	-0.023 ~ 0.156					1	3.30%
	JHJL	12									
Total	/	292	20	6.80%	0.040 ~ 0.097	5	1.70%	4	1.40%	11	3.80%
Dehong Prefecture	JGTA	30	3	10.00%	-0.007 ~ 0.207			2	6.70%	1	3.30%
	JGLS	30									
	JGYH	8									
	RLHP	30	1	3.30%	-0.023 ~ 0.156			1	3.30%		
	RLJK	30									
	RLPP	30									
	RLHF	30									
Total	/	188	4	2.10%	0.001 ~ 0.042	0	0.00%	3	1.60%	1	0.50%

### Phylogenetic analysis of *Wolbachia* in mosquitoes

**Figure 2:**
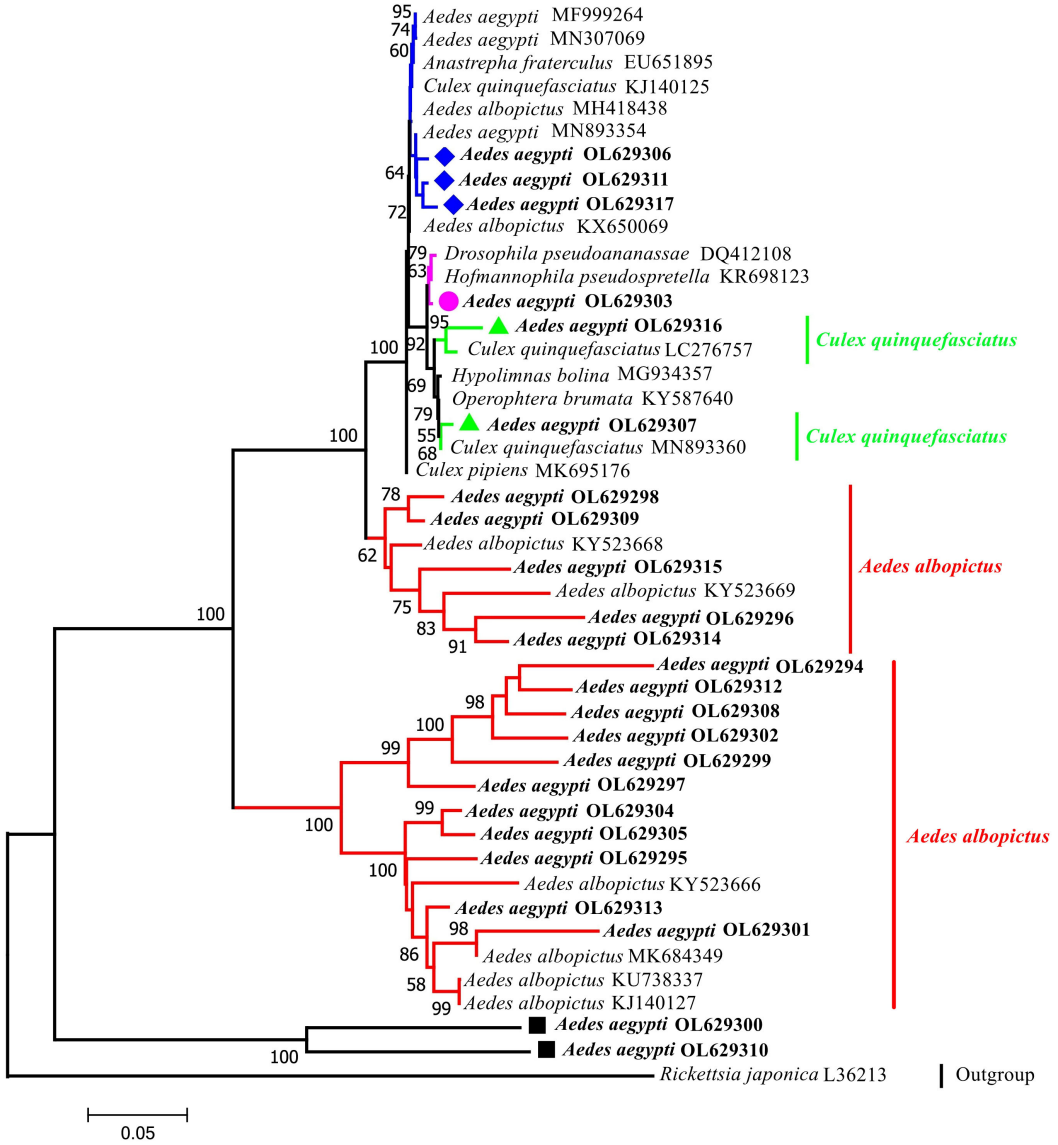
Phylogenetic tree analysis by the neighbor-joining method using the *wsp* gene (universal primers: 81F,691R). The tree contains 44 nucleotide sequences. 24 from this study; 19 from GenBank searches; 1 outgroup reference sequence (*Rickettsia japonica*) with Bootstrap values (1000 replicates) marked next to the branches. Taxa were tagged to obtain the host name of the *Wolbachia* strain and the ID in GenBank. the *wsp* sequences in this study all start with *Aedes aegypti* OL. OL629294 to OL629298 belong to JHGZ, Xishuangbanna prefecture. OL629299 to OL629301 belong to JHDM, Xishuangbanna prefecture. OL629302 and OL629303 belong to JHFZ, Xishuangbanna prefecture. OL629304 and OL629305 belong to JHML, Xishuangbanna prefecture. OL629306 and OL629307 belong to JHML, Xishuangbanna prefecture. OL629308 to OL629310 belong to DLAA, Xishuangbanna prefecture. OL629311 and OL629312 belong to DLXX, Xishuangbanna prefecture. OL629313 belongs to JHJL, Xishuangbanna prefecture. OL629314 to OL629316 belong to JGLS, Dehong prefecture. OL629317 belongs to RLJK, Dehong prefecture. The reference sequences used are shown in **Table S1**.

A phylogenetic tree was constructed using 44 sequences: 24 sequences collected in this study and 20 GenBank sequences (**Figure 2**). The phylogenetic analysis indicated that the outgroup (*Rickettsia japonica*) was independent of one branch. The target taxon was divided into two major branches with bootstrap values of 100%, which indicates that two major branches are plausible. The larger branches were further divided into four subbranches (bootstrap values of 100%, 62%, 99%, and 100%; thus, all four subbranches were plausible). Sixteen sequences (highlighted in red) were from the same node as the *Wolbachia* strains isolated in *Ae. albopictus* hosts and had a recent common ancestor. Samples YA10-4, YA17-13, YA8-28, YA5-3, YA14-21, and YA8-30 were from the same node and had high homology (bootstrap value of 100% indicating high support). The *Wolbachia* strains in these samples were relatively diverse in ancestry and may have shared a recent common ancestor with the *Wolbachia* strains found in *Cx. quinquefasciatus*, *Ae. aegypti*, *Drosophila pseudoananassae*, and *Hofmannophila pseudospretella*. Samples YA3-4 and YA9-25 formed a separate branch.

**Figure 3:**
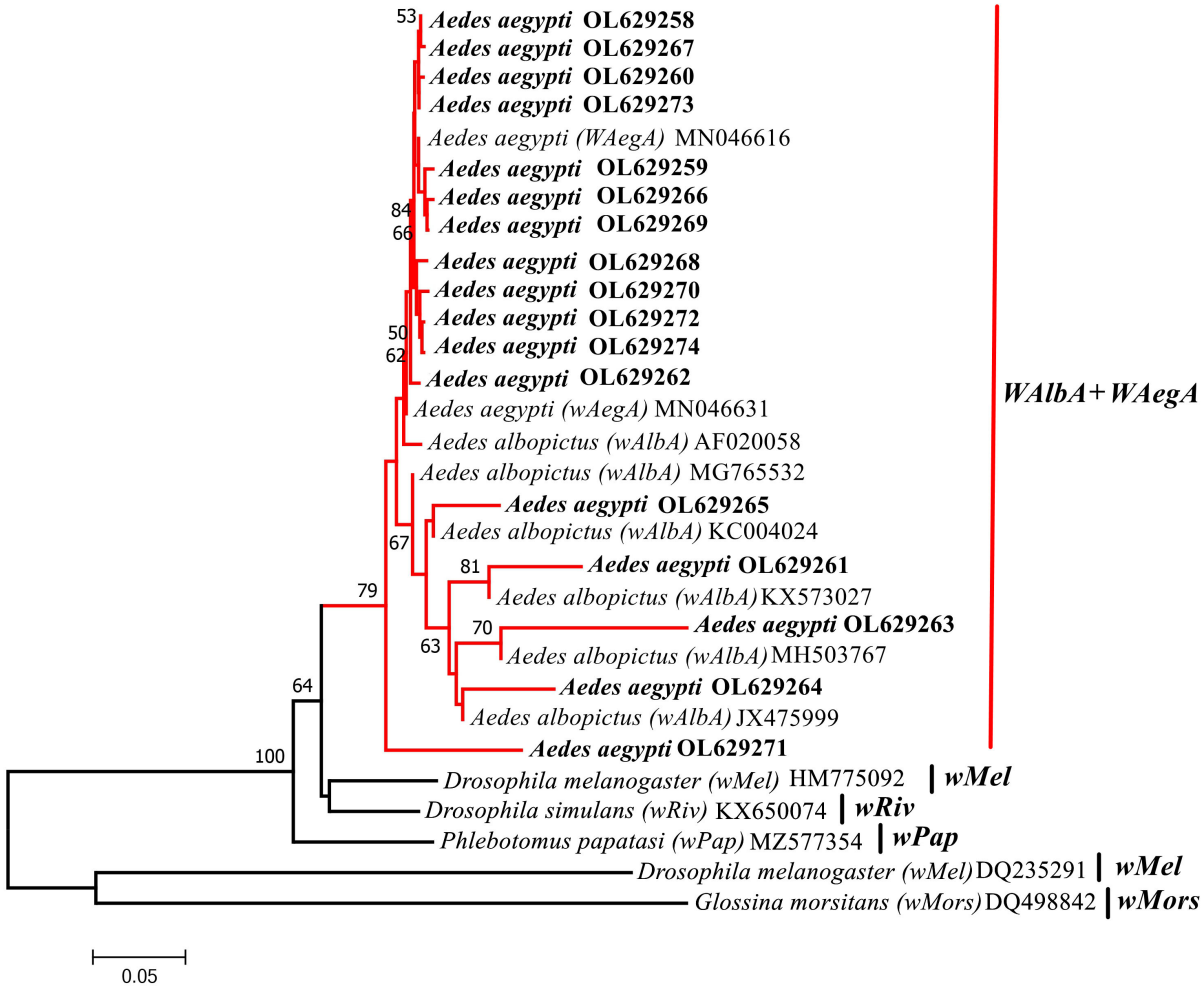
Phylogenetic tree analysis by the neighbor-joining method using the *wsp* gene (A supergroup primers: 136F,691R). The tree contains 30 nucleotide sequences. 17 from this study and 13 from GenBank searches with Bootstrap values (1000 replicates) marked next to the branches. Taxa were tagged to obtain the host name of the *Wolbachia* strain and the ID in GenBank. the *wsp* sequences in this study all start with *Aedes aegypti* OL. OL629258 to OL629262 belong to JHGZ, Xishuangbanna prefecture. OL629263 to OL629265 belong to JHDM, Xishuangbanna prefecture. OL629266 belongs to JHFZ, Xishuangbanna prefecture. OL629267 and OL629268 belong to JHML, Xishuangbanna prefecture. OL629269 to OL629271 belong to DLAA, Xishuangbanna prefecture. OL629272 belongs to DLXX, Xishuangbanna prefecture. OL629273 belongs to JHJL, Xishuangbanna prefecture. OL629274 belongs to JGLS, Dehong prefecture. The reference sequences used are shown in **Table S2**.

In addition, the sequences obtained with the Group A primers (*wsp*136F, *wsp*691R) were used to conduct a phylogenetic analysis. The phylogenetic analysis was performed by downloading multiple substrain reference sequences (*wAlbA*, *wAegA*, *wPap*, *wMel*, *wRiv*, and *wMors*) from Group A on the NCBI website. The results indicated that the phylogenetic tree had two branches (bootstrap value of 100%); the large branch yielded three subbranches. The *Ae. aegypti* sample from Yunnan Province (YA series), and the *wAlbA* and *wAegA* strains shared a recent common ancestor on one subbranch (**Figure 3**).

**Figure 4:**
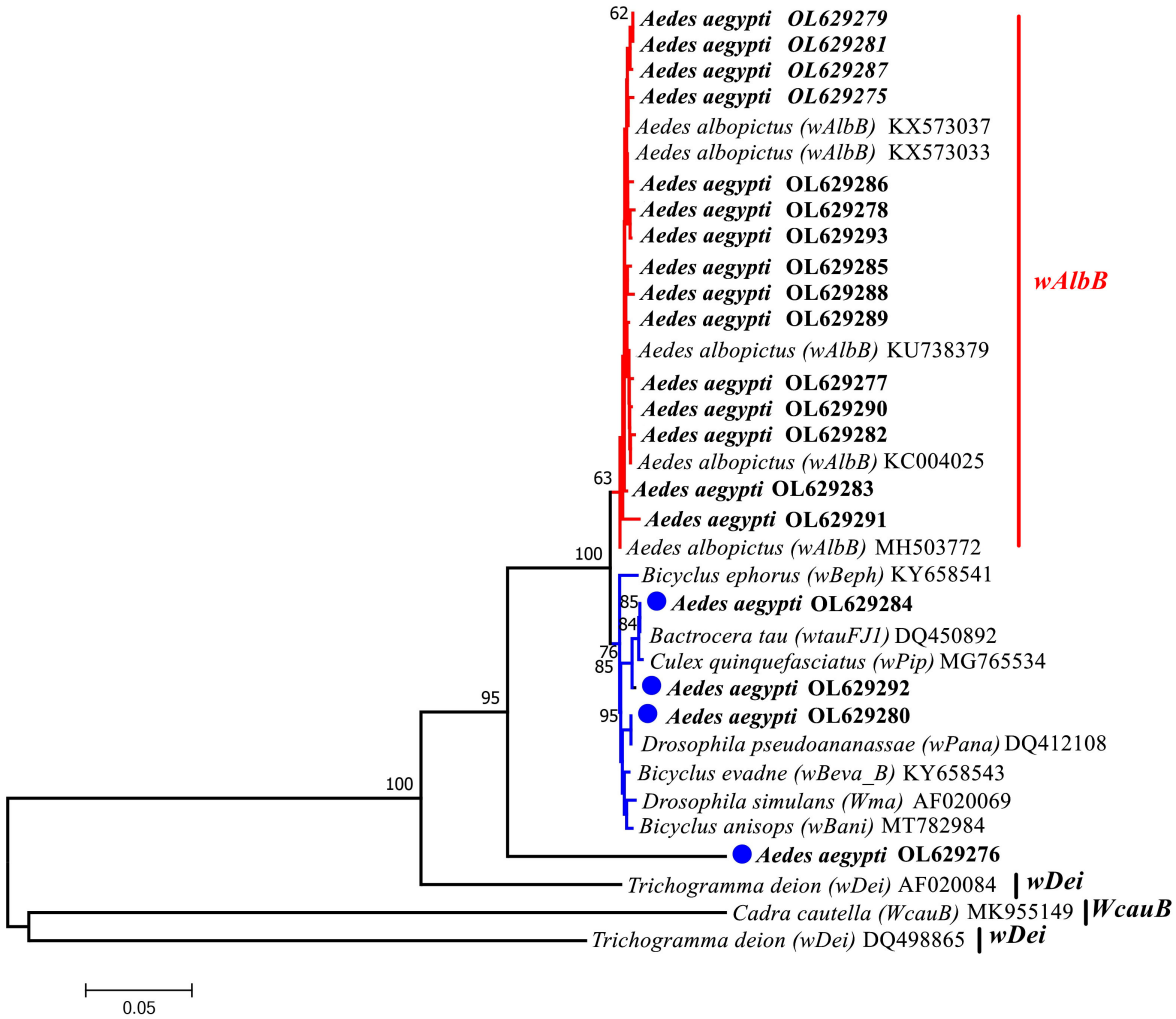
Phylogenetic tree analysis by the neighbor-joining method using the *wsp* gene (B supergroup primers: 183F,691R). The tree contains 34 nucleotide sequences. 19 from this study and 15from GenBank searches with Bootstrap values (1000 replicates) marked next to the branches. Taxa were tagged to obtain the host name of the *Wolbachia* strain and the ID in GenBank. the *wsp* sequences in this study all start with *Aedes aegypti* OL. OL629275 to OL629278 belong to JHGZ, Xishuangbanna prefecture. OL629279 and OL629280 belong to JHFZ, Xishuangbanna prefecture. OL629281 and OL629282 belongs to JHML, Xishuangbanna prefecture. OL629283 and OL629284 belong to JHGL2, Xishuangbanna prefecture. OL629285 and OL629286 belong to DLAA, Xishuangbanna prefecture. OL629287 and OL629288 belong to DLXX, Xishuangbanna prefecture. OL629289 belongs to JHJL, Xishuangbanna prefecture. OL629290 to OL629292 belong to JGLS, Dehong prefecture.OL629293 belongs to RLJK, Dehong prefecture. The reference sequences used are shown in **TableS3**.

In addition, the sequences obtained with the Group B primers (*wsp*183F, *wsp*691R) were used to conduct a phylogenetic analysis. The phylogenetic analysis of multiple substrain reference sequences of Group B (*wAlbB*, *wtauFJ1*, *wPip*, *wPana*, *wBeph*, *wBeva_B*, *Wma*, *wBani*, *wDei*, *WcauB*) downloaded from the NCBI website indicated that among the *Ae. aegypti* samples from Yunnan Province (YA series), 15 sequences (78.9%) were from the same node as the *wAlbB* strain and shared a recent common ancestor with the *Wolbachia* strain found in *Ae. albopictus*. In Clade 2, samples YA8-30-B, YA14-21-B and YA5-3-B were related to the *wtauFJ1*, *wPip*, and *wPana* strains, respectively, and clustered into a single strain. Sample YA2-2-B represented a separate strain (**Figure 4**).

This study also recorded the temperature in the sampled areas in 2018 to facilitate subsequent analysis. The diurnal temperature difference in Dehong Prefecture and Xishuangbanna Prefecture was in the range of 15–20°C. Except for Menghai County (MH), the average high temperature was above 26°C and the highest temperatures were above 28°C. Data were provided by the Global Weather Network (www.tianqi.com) (**Table 1**).

**Figure 5:**
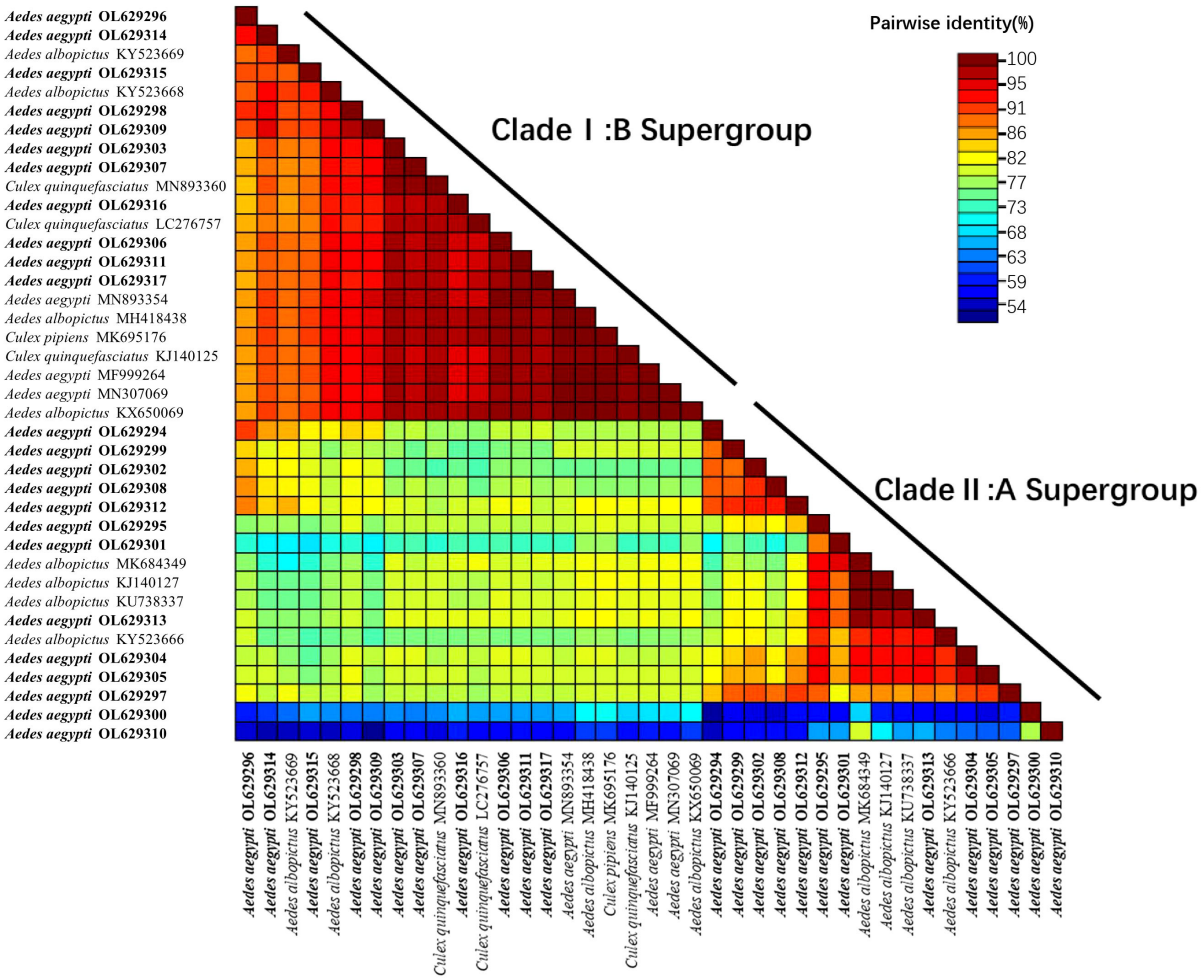
Heat map of sequence similarity between sequences obtained using the *wsp* gene (universal primers: w81F, w691R) and *Wolbachia* sequences of different mosquito species. The heat map contains 39 nucleotide sequences (24 from this study; 15 from GenBank search), the 15 sequences from GenBank include: 3 from *Ae. aegypti*, 8 from *Aedes albopictus*, 3 from *Cx.pipiens quinquefasciatus* and 1 from *Cx pipiens*. The number of reference sequences for these four mosquitoes was chosen based on random selection of sequences with higher homology during homology matching on NCBI. Clustal W alignment were performed for each unique pair of sequences, pairwise similarity scores were calculated, and a color-coded matrix of these scores is displayed. The *wsp* sequences in this study all start with *Aedes aegypti* OL. OL629294 to OL629298 belong to JHGZ, Xishuangbanna prefecture. OL629299 to OL629301 belong to JHDM, Xishuangbanna prefecture. OL629302 and OL629303 belong to JHFZ, Xishuangbanna prefecture. OL629304 and OL629305 belong to JHML, Xishuangbanna prefecture. OL629306 and OL629307 belong to JHML, Xishuangbanna prefecture. OL629308 to OL629310 belong to DLAA, Xishuangbanna prefecture. OL629311 and OL629312 belong to DLXX, Xishuangbanna prefecture. OL629313 belongs to JHJL, Xishuangbanna prefecture. OL629314 to OL629316 belong to JGLS, Dehong prefecture. OL629317 belongs to RLJK, Dehong prefecture.

In this study, a heatmap of sequence similarity was generated based on the *wsp* sequences obtained from the universal primers (**Figure 5**). The heatmap included *Wolbachia* sequences from four mosquito species and was divided into the Clade I:B supergroup and Clade II:A supergroup. In the Clade I:B supergroup, the sequences identified in this study were highly homologous to those of *Wolbachia* found in *Ae. aegypti* in Malaysia (ID: MN893354) (Wong et al., 2020) and India (ID: MN307069 and MF999264). These sequences were not comparable to the *Wolbachia* found in *Ae. aegypti* from the United States (Hegde et al., 2018; Kulkarni et al., 2019); thus, this comparison is not presented on the heatmap. The sequences were also highly homologous with those found in *Cx. pipiens quinquefasciatus*, *Cx. pipiens* and *Ae. albopictus*, but the homology with those found in *Ae. albopictus* was more easily detected. In Clade II:A supergroup, all sequences had high homology with the *Wolbachia* found in *Ae. albopictus*.

## Discussion

This study is the first to report that populations of *Ae. aegypti* in Yunnan Province, China were naturally infected with *Wolbachia*. The average infection rate of *Wolbachia* in *Ae. aegypti* populations was 5%, higher than that of Florida (reported at 4.3%) and lower than that of the Philippines (11%) (Carvajal et al., 2019; Kulkarni et al., 2019). Compared with the infection rate in *Ae. albopictus* and *Cx. pipiens*, the infection rate of *Wolbachia* in *Ae. aegypti* was lower, which may be related to the environmental temperatures and lower density of *Wolbachia* in *Ae. aegypti* in the wild. Densities of *Wolbachia* in *Ae. albopictus* tended to decrease with increasing temperature, and *Wolbachia* endosymbionts could be removed from the host by exposure to heat or antibiotics (Hermans et al., 2001; Lau et al., 2020). In China, *Ae. aegypti* is more concentrated in tropical and subtropical regions than *Ae. albopictus* and *Cx.* spp., and the temperature of their habitat was higher than that of *Ae. albopictus* and *Cx*. spp. Rearing *Wolbachia*-infected larvae at 26-37°C reduced the rates of cytoplasmic incompatibility and dramatically decreased the density of *Wolbachia* in adult mosquitoes. Experiments on the response of *Ae. aegypti* infected with *Wolbachia* to cyclical heat stress have suggested that the likelihood of *Wolbachia* invasion and persistence in populations depends on interactions with environmental conditions, particularly the exposure of larvae to frequent temperature fluctuations and extremes (Ross et al., 2017). In 2018, the average highs and highest temperatures in Dehong Prefecture and Xishuangbanna Prefecture were in the range of 26–37°C (**Table 1**); thus, the low infection rate of *Wolbachia* in *Ae. aegypti* may be caused by the environmental temperatures.

Competition among co-infected microorganisms may result in a decrease in *wolbachia* titer. *Wolbachia* usually co-exists with other endosymbiotic bacteria or members of the gut microbiota (Gómez-Valero et al., 2004; Dittmer et al., 2016). Co-infection of multiple bacterial lineages might translate into competition for space and nutrients (Caragata et al., 2014; Geoghegan et al., 2017; Jiménez et al., 2019). The difference in intestinal microbial composition between *Ae. aegypti* and other mosquitoes resulted in high competition and low titer of Wolbachia. *Ae. aegypti* ‘s own immune response plays an important role (Masson et al., 2016). *Wolbachia* is known to activate the basal immune response of *Ae. aegypti via* the immune deficiency (IMD) and Toll-pathway. Silencing of these immune pathways leads to the reduction of *Wolbachia* titers (Pan et al., 2018).

*Ae. aegypti* did not occur in Yunnan Province before 2000, as this species was first found in Ruili Port in 2002 (Dong Xue-shu, 2004). Since then, *Ae. aegypti* has been found in Mangshi, Mengla, Menghai, Jinghong, and other places in Yunnan Province (Shi et al., 2017). This suggests that *Ae. aegypti* is an important invasive alien mosquito species in Yunnan Province. Dehong Prefecture borders Myanmar and contains the largest China-Myanmar port, Ruili. Xishuangbanna Prefecture borders Laos and contains the largest China-Laos port, Mohan. *Ae. aegypti* populations in Southeast Asia may thus invade Yunnan Province from border ports through logistics and the movement of people. The results of this study thus provide valuable evidence for analysing the invasion of *Ae. Aegypti* in Yunnan Province. The time sequence of *Ae. aegypti* monitoring reports from different areas of Yunnan Province indicates that *Ae. aegypti* mosquitoes in Dehong Prefecture and Xishuangbanna Prefecture originated from separate invasion events. This implies a continuous invasion in different locations based on the types and rates of *Wolbachia* infection of *Ae. aegypti* populations in the area.

According to the phylogenetic tree results, all *Wolbachia* Group A (17/17) and 78.9% of *Wolbachia* Group B (15/19) infections in *Ae. aegypti* in this study clustered with *wAlbA* and *wAlbB* strains isolated from *Ae. albopictus*. The *Wolbachia* strains in *Ae. aegypti* were mainly classified as *wAlbA* and *wAlbB* (Coon et al., 2016; Carvajal et al., 2019; Wong et al., 2020), but the detection rates of *wAlbB* and *wAlbA* strains in *Ae. aegypti* differed. In India and the United States, no Group A infections have been found and only Group B was found, which is closely related to the *wAlbB* strain isolated from *Ae. Albopictus* (Balaji et al., 2019; Kulkarni et al., 2019). Both Group A and Group B were found in *Ae. aegypti* in China and the Philippines, but the infection rates of these strains differed. Of the Philippines samples, 60.7% (51/84) were clustered with *wAlbB* and all (29/29) samples clustered with *wAlbA* (Carvajal et al., 2019).

The sequence similarity heatmap of the *wsp* sequences obtained from the universal primers (**Figure 5**) was split into the Clade I:B and Clade II:A supergroups. In the Clade I:B supergroup, the sequences collected in this study were highly homologous to those of *Wolbachia* found in *Ae. aegypti* in Malaysia (ID: MN893354) (Wong et al., 2020) and India (ID: MN307069 and MF999264) but cannot be compared to the *Wolbachia* sequences in *Ae. aegypti* in the United States as Group B is not found in this region (Hegde et al., 2018; Kulkarni et al., 2019); thus, this comparison is not presented in the heatmap. The sequences collected in this study were also highly homologous to those of *Wolbachia* in *Cx. pipiens quinquefasciatus*, *Cx. pipiens* and *Ae. albopictus*, but this strain of *Wolbachia* occurs more frequently in *Ae. albopictus*. In Clade II:A supergroup, all sequences were highly homologous to those of *Wolbachia* found in *Ae. albopictus*. Comparing the strains found within the same species (*Ae. aegypti)*, the *Wolbachia* sequence found in this study had high homology with the *Wolbachia* sequence in *Ae. aegypti* distributed in countries that are geographically close to China and low homology with geographically distinct populations, such as the *Wolbachia* sequence in *Ae. aegypti* distributed in the United States. Comparing the strains found in different mosquitoes, the *Wolbachia* sequences in *Ae. aegypti* were highly homologous to those in *Ae. Albopictus*.

Natural infection of *Wolbachia* is found rarely in *Ae. aegypti*. Currently, studies on natural infection of *Ae. aegypti* with *Wolbachia* have only been reported in Malaysia, India, Thailand, the Philippines, and the U.S. states of Mexico and Florida. Studies of natural infection of *Ae. aegypti* with *Wolbachia* on reproduction and physiology and its efficacy on vectors have not been reported and further studies are needed (Teo et al., 2017; Thongsripong et al., 2018; Balaji et al., 2019; Carvajal et al., 2019; Kulkarni et al., 2019).Although not naturally found in *Ae. aegypti*, *wMel* strain were stably introduced into this mosquito in 2011 and were shown to reduce the transmission potential of dengue, Zika and chikungunya (Moreira et al., 2009; Walker et al., 2011; Aliota et al., 2016). *Ae. aegypti* carrying the *wMel* or *wAlbB* strains of *Wolbachia* have the potential to reduce dengue transmission through decreased mosquito vector competence, and there is already good evidence that both strains are having such impacts in *Wolbachia*-invaded release areas. In 2021, researchers found that long-term storage under warm environment greatly reduces the fertility of hatched females, especially for *wAlbB*-infected, in which a high proportion of females became infertile (Lau et al., 2021). Prevalence of *Ae. aegypti* infection with *Wolbachia* is found in an area, the impact of this situation on arbovirus transmission as well as vector control needs to be considered. Natural infection of *Wolbachia* by *Ae. albopictus* and a study showing that naturally occurring strains of *Wolbachia* can also restrict salivary gland infection of *Ae. albopictus* with DENV and limit transmission (Mousson et al., 2012; Ciota, 2019). *Wolbachia* strain *wMel* transfected into *Ae. albopictus* can induce cytoplasmic incompatibility and block dengue transmission in *Ae. Albopictus* (Blagrove et al., 2012). *Ae. albopictus* is currently facing such a situation, and it seems that the artificial release of *Ae. albopictus* infected with *Wolbachia* is also effective in reducing the density of *Ae. albopictus* already naturally infected with *Wolbachia (* Zheng et al., 2019 *)*.

## Conclusions

This was the first study to report *Wolbachia* infection in wild *Ae. aegypti* caught in China. *Wolbachia* was detected in wild populations of this species in Dehong and Xishuangbanna Prefectures of Yunnan Province. A total of 24 mosquitoes (5%) infected with *Wolbachia* were detected using *wsp* markers. The strain had high homology with *wAlbA* and *wAlbB*, and was prevalent in the wild population of *Ae. aegypti* in Yunnan Province. This study provides a basis for studying natural *Wolbachia* infections in wild populations of *Ae. aegypti*.

## Data availability statement

The datasets presented in this study can be found in online repositories. The names of the repository/repositories and accession number(s) can be found below: The data presented in the study are deposited in the GenBank database, accession numbers ON637917 to ON637937, OL629258 to OL629317.

## Author contributions

HZ, JG, ZM, XG, and TYZ jointly designed and coordinated the study, with contributions from CL, YTD, DX and YDD. HZ and ZM drafted the article with contributions from JG. HZ, JG, YL, GW, QL, YJ, and TZ collected samples from Yunnan Province of China. HZ, JG, and ZM carried out the laboratory work and performed the statistical analysis. All authors contributed to the article and approved the submitted version.

## Funding

This work was funded by grants from the Infective Diseases Prevention and Cure Project of China (No.2017ZX10303404).

## Conflict of interest

The authors declare that the research was conducted in the absence of any commercial or financial relationships that could be construed as a potential conflict of interest.

## Publisher’s note

All claims expressed in this article are solely those of the authors and do not necessarily represent those of their affiliated organizations, or those of the publisher, the editors and the reviewers. Any product that may be evaluated in this article, or claim that may be made by its manufacturer, is not guaranteed or endorsed by the publisher.
