# Supplementary material for: Wolbachia infection in field-collected Aedes aegypti in Yunnan Province, southwestern China
Source: Front Cell Infect Microbiol. 2022 Nov 30;12:1082809. doi: 10.3389/fcimb.2022.1082809 (PMC9748079; doi:10.3389/fcimb.2022.1082809)
Supplement: Supplementary file 1 [file Table_1.docx]

Supplementary Material

# Supplementary Table

| Table S1 Sequences of different host *Wolbachia* strains based on *wsp* molecular markers | | | |
| --- | --- | --- | --- |
| Subgroup | Strain | Natural *Wolbachia* host | GenBank ID |
|  |  | *Aedes aegypti* | MF999264 |
|  |  | *Aedes aegypti* | MN307069 |
|  |  | *Aedes aegypti* | MN893354 |
|  |  | *Aedes albopictus* | MH418438 |
|  |  | *Aedes albopictus* | KX650069 |
|  |  | *Aedes albopictus* | KU738337 |
|  |  | *Aedes albopictus* | KJ140127 |
|  |  | *Aedes albopictus* | MK684349 |
|  |  | *Aedes albopictus* | KY523669 |
|  |  | *Aedes albopictus* | KY523668 |
|  |  | *Aedes albopictus* | KY523666 |
|  |  | *Culex quinquefasciatus* | KJ140125 |
|  |  | *Culex quinquefasciatus* | MN893360 |
|  |  | *Culex quinquefasciatus* | LC276757 |
|  |  | *Culex pipiens* | MK695176 |
|  |  | *Anastrepha fraterculus* | EU651895 |
|  |  | *Drosophila pseudoananassae* | DQ412108 |
|  |  | *Hypolimnas bolina* | MG934357 |
|  |  | *Hofmannophila pseudospretella* | KR698123 |
|  |  | *Operophtera brumata* | KY587640 |
|  | outgroup | *Rickettsia japonica* | L36213 |

| Table S2 Sequences of different host *Wolbachia* strains based on *wsp* molecular markers | | | |
| --- | --- | --- | --- |
| Subgroup A | Strain | Natural *Wolbachia* host | GenBank ID |
|  | *wAegA* | *Aedes aegypti* | MN046616 |
|  | *wAlbA* | *Aedes albopictus* | AF020058 |
|  | *wAlbA* | *Aedes albopictus* | MG765532 |
|  | *wAlbA* | *Aedes albopictus* | KC004024 |
|  | *wAlbA* | *Aedes albopictus* | JX475999 |
|  | *wAlbA* | *Aedes albopictus* | MH503767 |
|  | *wAlbA* | *Aedes albopictus* | KX573027 |
|  | *wAegA* | *Aedes aegypti* | MN046631 |
|  | *wMel* | *Drosophila melanogaster* | HM775092 |
|  | *wMel* | *Drosophila melanogaster* | DQ235291 |
|  | *wMors* | *Glossina morsitans* | DQ498842 |
|  | *wPap* | *Phlebotomus papatasi* | MZ577354 |
|  | *wRiv* | *Drosophila simulans* | KX650074 |

| Table S3 Sequences of different host *Wolbachia* strains based on *wsp* molecular markers | | | |
| --- | --- | --- | --- |
| Subgroup B | Strain | Natural *Wolbachia* host | GenBank ID |
|  | *wAlbB* | *Aedes albopictus* | KX573033 |
|  | *wAlbB* | *Aedes albopictus* | KC004025 |
|  | *wAlbB* | *Aedes albopictus* | KU738379 |
|  | *wAlbB* | *Aedes albopictus* | MH503772 |
|  | *wAlbB* | *Aedes albopictus* | KX573037 |
|  | *wBeph* | *Bicyclus ephorus* | KY658541 |
|  | *wBeva_B* | *Bicyclus evadne* | KY658543 |
|  | *wBani* | *Bicyclus anisops* | MT782984 |
|  | *wPana* | *Drosophila pseudoananassae* | DQ412108 |
|  | *Wma* | *Drosophila simulans* | AF020069 |
|  | *wDei* | *Trichogramma deion* | DQ498865 |
|  | *wDei* | *Trichogramma deion* | AF020084 |
|  | *WcauB* | *Cadra cautella* | MK955149 |
|  | *wtauFJ1* | *Bactrocera tau* | DQ450892 |
|  | *wPip* | *Culex quinquefasciatus* | MG765534 |
